# Therapeutic Plasma Exchange for Murray Valley Encephalitis: A Case Report and Review of Clinical Features, Diagnosis, and Challenges in Management

**DOI:** 10.7759/cureus.69696

**Published:** 2024-09-19

**Authors:** Hamish A Derrick, Matthew H Anstey, Penelope Clohessy, Tristan Gibbs

**Affiliations:** 1 Intensive Care, Sir Charles Gairdner Hospital, Perth, AUS; 2 Infectious Diseases, Sir Charles Gairdner Hospital, Perth, AUS; 3 Microbiology, Sir Charles Gairdner Hospital, Perth, AUS

**Keywords:** fatal, flair, flavivirus, infection, intensive care, mosquito vector, mri brain, murray valley encephalitis, therapeutic plasma exchange, western australia

## Abstract

We report the case of a 62-year-old male who presented to the Emergency Department (ED) with altered mental status following a motor vehicle accident. He was transferred to the Intensive Care Unit (ICU) with worsening of his neurological status and subsequently diagnosed with Murray Valley encephalitis: a serious but rare condition caused by infection with Murray Valley encephalitis virus (MVEV). He continued to deteriorate despite treatment with antivirals, glucocorticoids, and therapeutic plasma exchange (TPE) in addition to supportive care and eventually succumbed to his illness. In this report, we review the clinical course, pathophysiology, diagnosis, and characteristic radiological features of Murray Valley encephalitis and highlight the challenges in treating this potentially devastating disease.

## Introduction

Murray Valley encephalitis virus (MVEV) is a flavivirus endemic to northern Australia [1]. There is a broad spectrum of disease, with only a minority of infections causing symptoms. Both encephalitic and nonencephalitic cases have been described [1]. When symptomatic disease does occur, there is typically a prodrome of nonspecific symptoms such as lethargy, fever, and cough, which may be followed by recovery, or in a very small minority by development of neurological features such as seizure, movement disorder, flaccid paralysis, and cranial nerve palsy [1]. When neurological involvement is present, the case fatality rate is high [1]. Diagnosis of MVEV infection is by culture of the virus, detection by polymerase chain reaction (PCR), a fourfold rise in MVEV-specific IgG titer between two serum samples, or detection of IgM in cerebrospinal fluid (CSF) without detection of other flavivirus-specific IgM [2]. Characteristic findings on magnetic resonance imaging (MRI) include high T2-weighted signal intensities affecting the thalami bilaterally that progress to involve the brainstem as the disease progresses [3]. Treatment is supportive, as no therapeutic options have proven to be useful [1]. This is despite the high rates of fatality and permanent disability associated with the encephalitic form of the disease [1,3,4].

## Case presentation

A 62-year-old male with a history of ischemic heart disease and heavy alcohol use was brought to the Emergency Department (ED) by police following a low-speed motor vehicle accident. The patient’s spouse reported that he had appeared disoriented and confused that morning and had been experiencing flulike symptoms for two weeks prior. He had travelled to Karratha in the Pilbara region of northern Western Australia (WA) in the weeks preceding his illness.

In the ED, the patient was found to be febrile, tachycardic, and disoriented. There were no signs of meningism. Blood work on admission is shown in Table 1. White cell count and C-reactive protein were within normal limits. Liver function tests revealed elevated levels of gamma-glutamyl transferase and alanine aminotransferase. Renal function tests were unremarkable, other than for mild hyponatremia. He became increasingly agitated and required intubation to facilitate a computed tomography (CT) scan which did not demonstrate any acute intracranial abnormality. He was commenced on empirical acyclovir, ceftriaxone, and dexamethasone to cover for viral encephalitis and bacterial meningitis.

**Table 1:** Blood work on admission WCC: White cell count; Hb: hemoglobin; CRP: C-reactive protein; ALT: alanine aminotransferase; ALP: alkaline phosphatase; GGT: gamma-glutamyl transferase

Parameter	Value	Reference range
WCC	9.54 x 10^9^/L	4.00-11.00 x 10^9^/L
Hb	156 g/L	135-180 g/L
Platelet count	119 x 10^9^/L	150-400 x 10^9^/L
CRP	<1.0 mg/L	<5.0 mg/L
Bilirubin	19 umol/L	<20 umol/L
ALT	45 U/L	<40 U/L
ALP	43 U/L	30-110 U/L
GGT	227 U/L	<60 U/L
Sodium	134 mmol/L	135-145 mmol/L
Potassium	3.6 mmol/L	3.5-5.2 mmol/L
Urea	5.3 mmol/L	3.0-8.0 mmol/L
Creatinine	101 umol/L	60-110 umol/L

**Figure 1:**
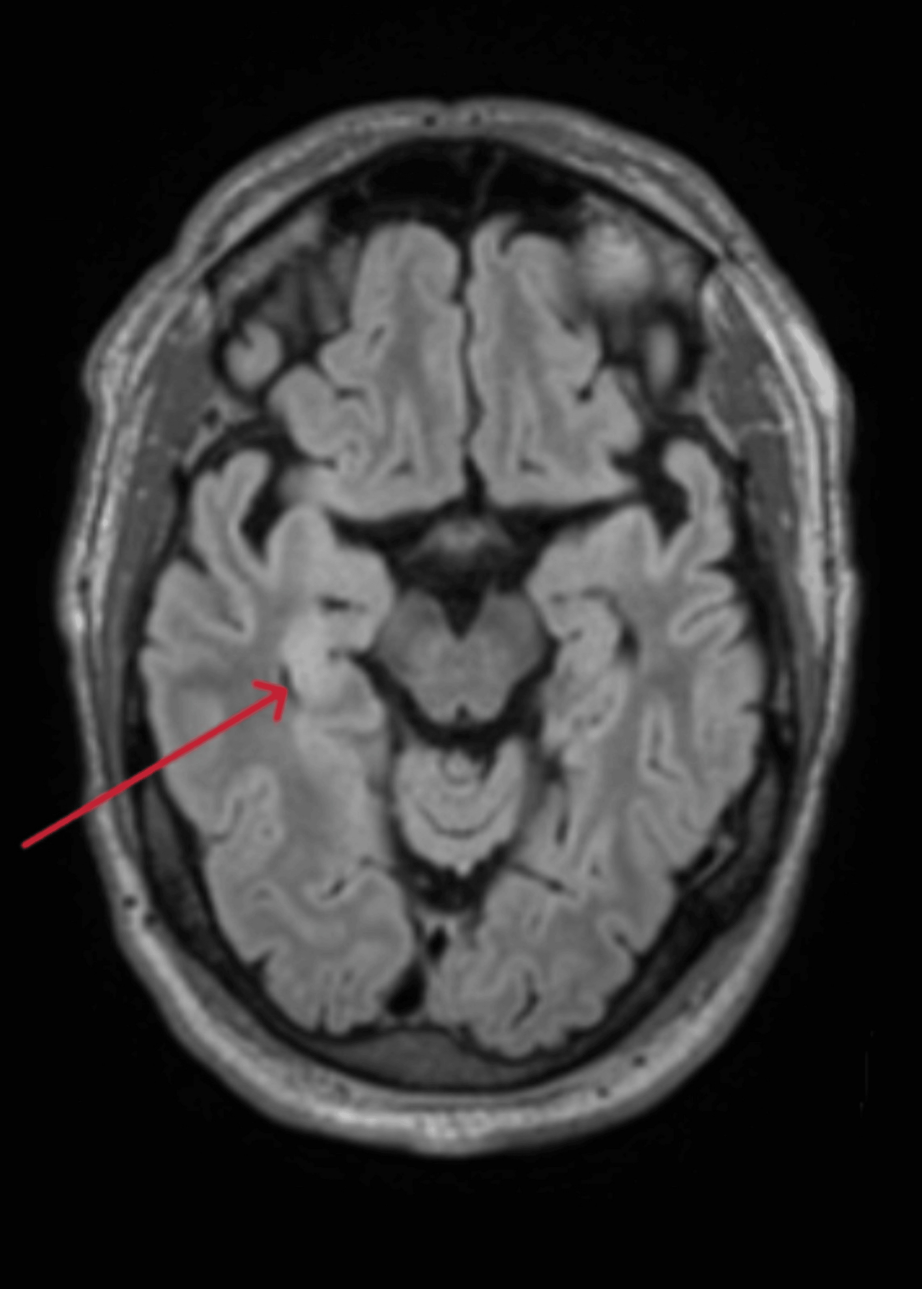
Axial brain MRI showing increased FLAIR signal in the right hippocampus MRI: Magnetic resonance imaging; FLAIR: fluid-attenuated inversion recovery

**Figure 2:**
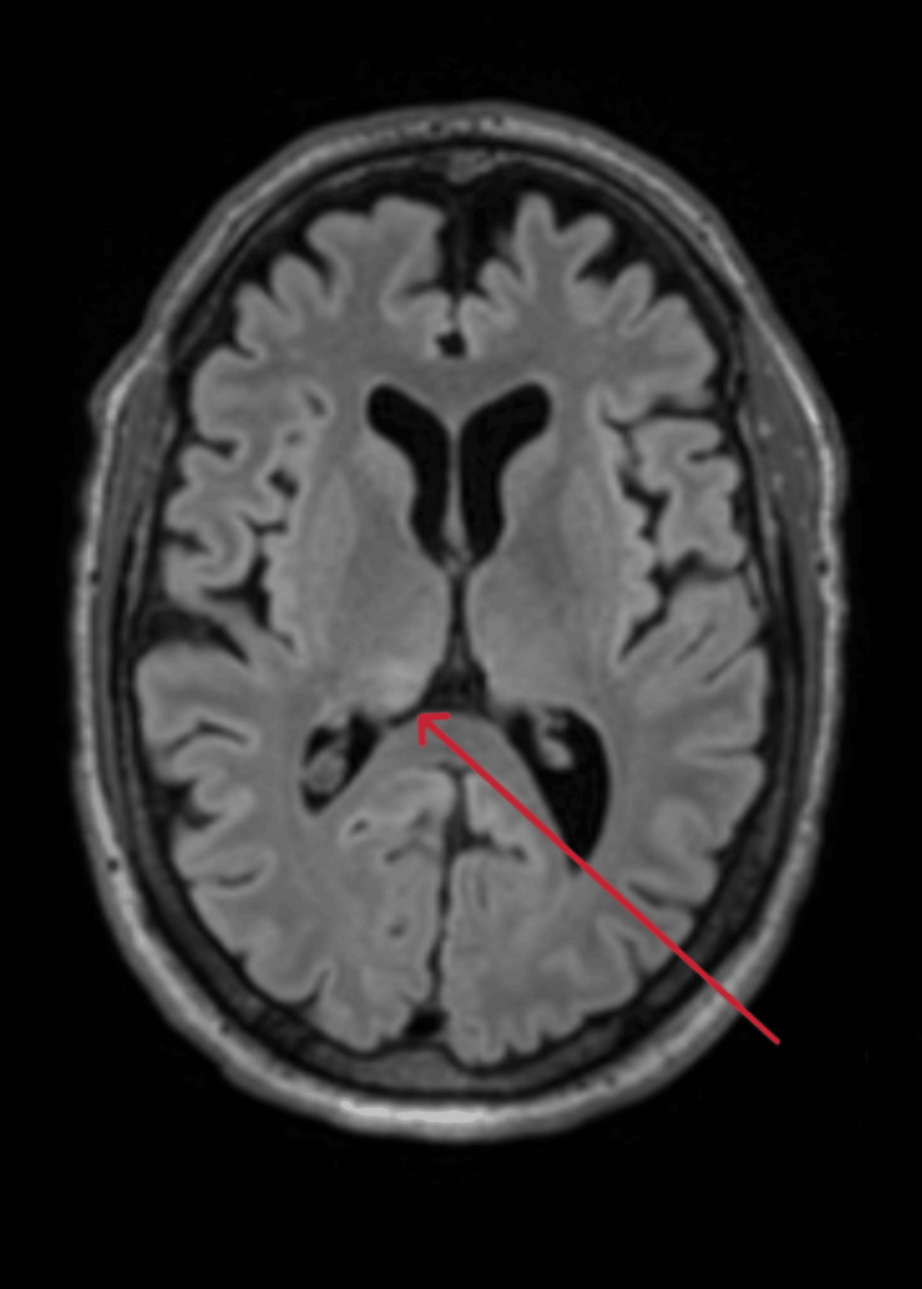
Axial brain MRI showing increased FLAIR signal in the right thalamus MRI: Magnetic resonance imaging; FLAIR: fluid-attenuated inversion recovery

He was subsequently transferred to the Intensive Care Unit (ICU) for ongoing care, where benzylpenicillin was added into his treatment regimen to cover for *Listeria monocytogenes*, given his age and alcohol use. An MRI of his brain demonstrated increased fluid-attenuated inversion recovery (FLAIR) signal in the right hippocampus and thalamus in keeping with a diagnosis of encephalitis (Figures 1, 2). Lumbar puncture (LP) was delayed until two days into ICU admission, due to the patient being on antiplatelet therapy. CSF was colorless, with mildly elevated glucose and protein (Table 2). Analysis of CSF included microscopy, culture, and sensitivity, and PCR and serology for viral, bacterial, and fungal causes of encephalitis, and a screen for autoimmune and paraneoplastic causes.

**Table 2:** CSF findings from first LP CSF: Cerebrospinal fluid; LP: lumbar puncture

Parameter	Value	Reference range
Glucose	6.3 mmol/L	2.7-4.4 mmol/L
Protein	0.60 g/L	0.15-0.45 g/L
Leukocyte count	3 x 10^6^/L	≤5 x 10^6^/L
Lactate	2.6 mmol/L	1.4-2.6 mmol/L
Pyruvate	0.08 mmol/L	0.05-0.16 mmol/L
Tau protein	720 ng/L	≤536 ng/L
14-3-3 protein	111,297 AU/mL	≤23,194 AU/mL

Four days into admission, an infective or autoimmune cause had not been identified, and dexamethasone and antibiotics were ceased. Further blood was collected for analysis, which included arbovirus serology. An electroencephalogram was consistent with severe encephalopathy. The patient’s neurology deteriorated, and on his fifth day of ICU admission, he had an absent cough and gag reflex and no response to pain. Regular levetiracetam was commenced, and acyclovir was continued to cover for herpes simplex encephalitis. Following an unremarkable repeat CT head scan, LP was repeated and further CSF sent for analysis.

Antibody testing on serum was provisionally reported as being positive for IgM against MVEV on the seventh day of admission. By this stage, given the continued decline in the patient’s neurological status in the absence of any clear cause, a trial of intravenous methylprednisolone and therapeutic plasma exchange (TPE) had been suggested. A first dose of methylprednisolone was given prior to the antibody testing result being confirmed the following day. At this stage, CSF from the second LP also returned a positive result for MVEV-specific IgM, and consequently acyclovir was ceased.

In the absence of any specific treatments for MVEV, we continued daily methylprednisolone for a total of three days and started TPE based on case reports of its successful use for the treatment of other flaviviruses [5,6]. His admission was further complicated by ventilator-associated pneumonia, for which he was recommenced on intravenous antibiotics, and fluid overload requiring diuretics. Incidentally, CSF from the patient’s first LP had by this time been reported as being positive for the Tau and 14-3-3 protein biomarkers for Creutzfeldt-Jakob disease. Real-time quaking-induced conversion (RT-QuIC) was negative.

**Figure 3:**
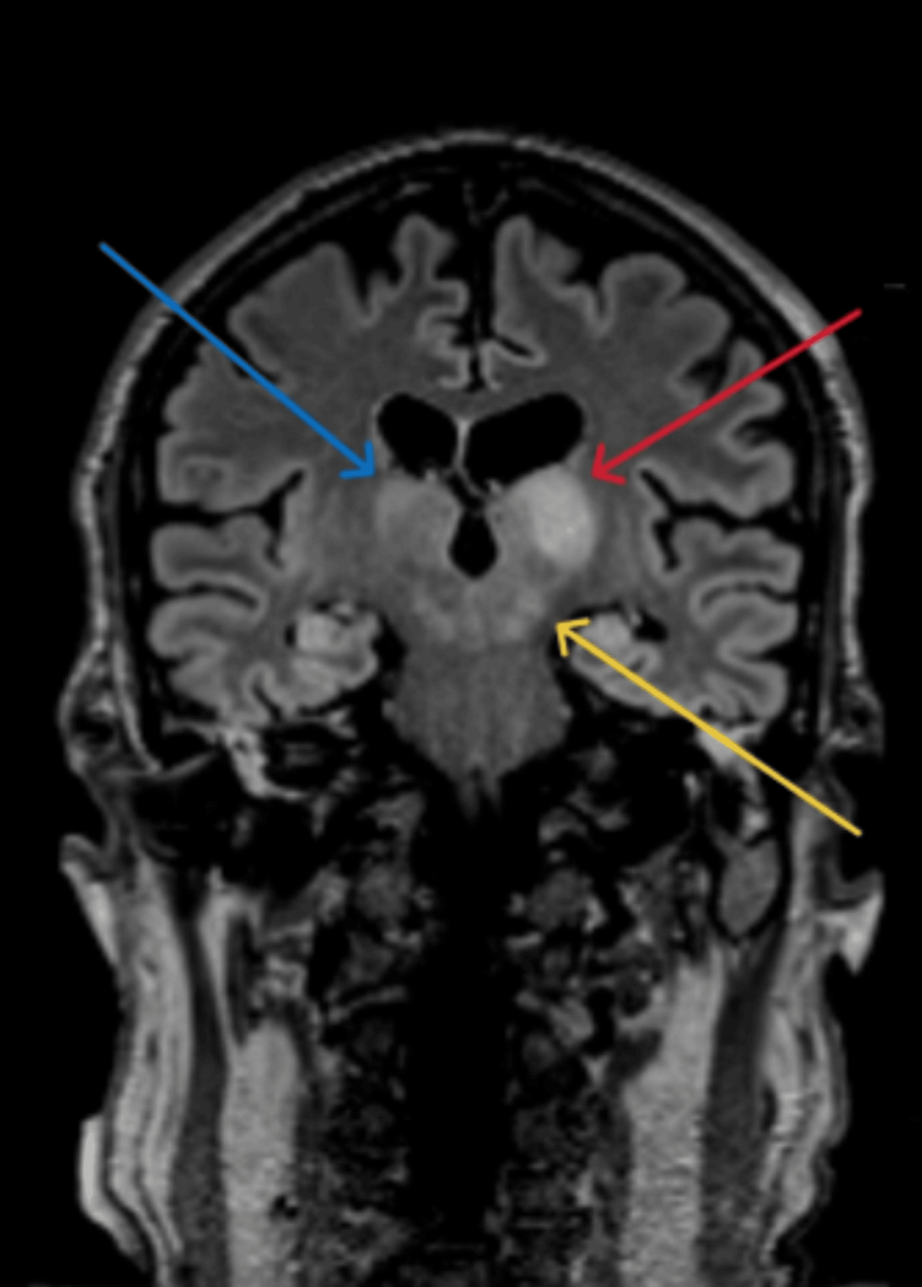
Coronal brain MRI showing increased FLAIR signal in the left thalamus (red arrow), lateral right thalamus (blue arrow), and midbrain (yellow arrow) MRI: Magnetic resonance imaging; FLAIR: fluid-attenuated inversion recovery

**Figure 4:**
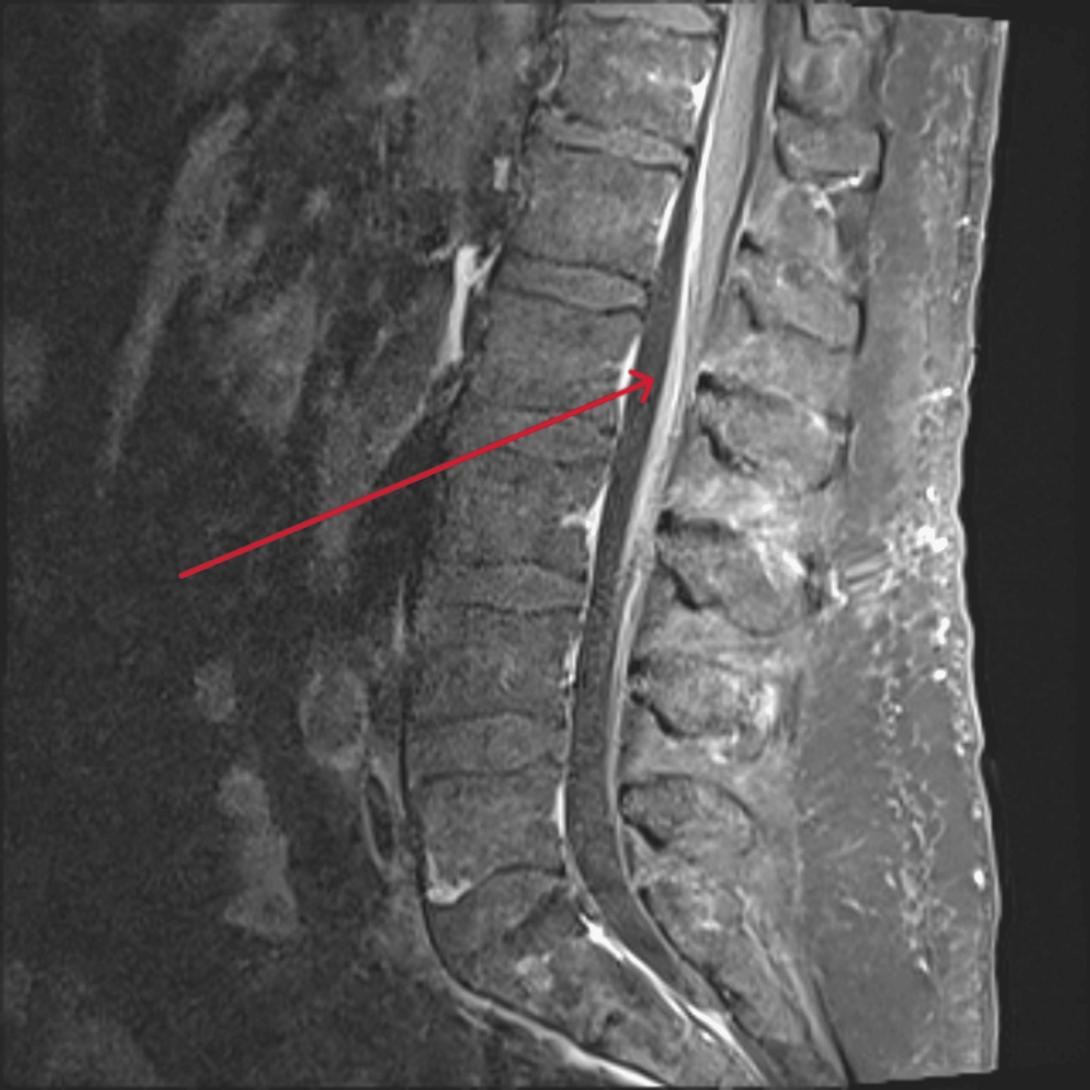
MRI showing abnormal enhancement of ventral rootlets in the lumbar spine MRI: Magnetic resonance imaging

**Figure 5:**
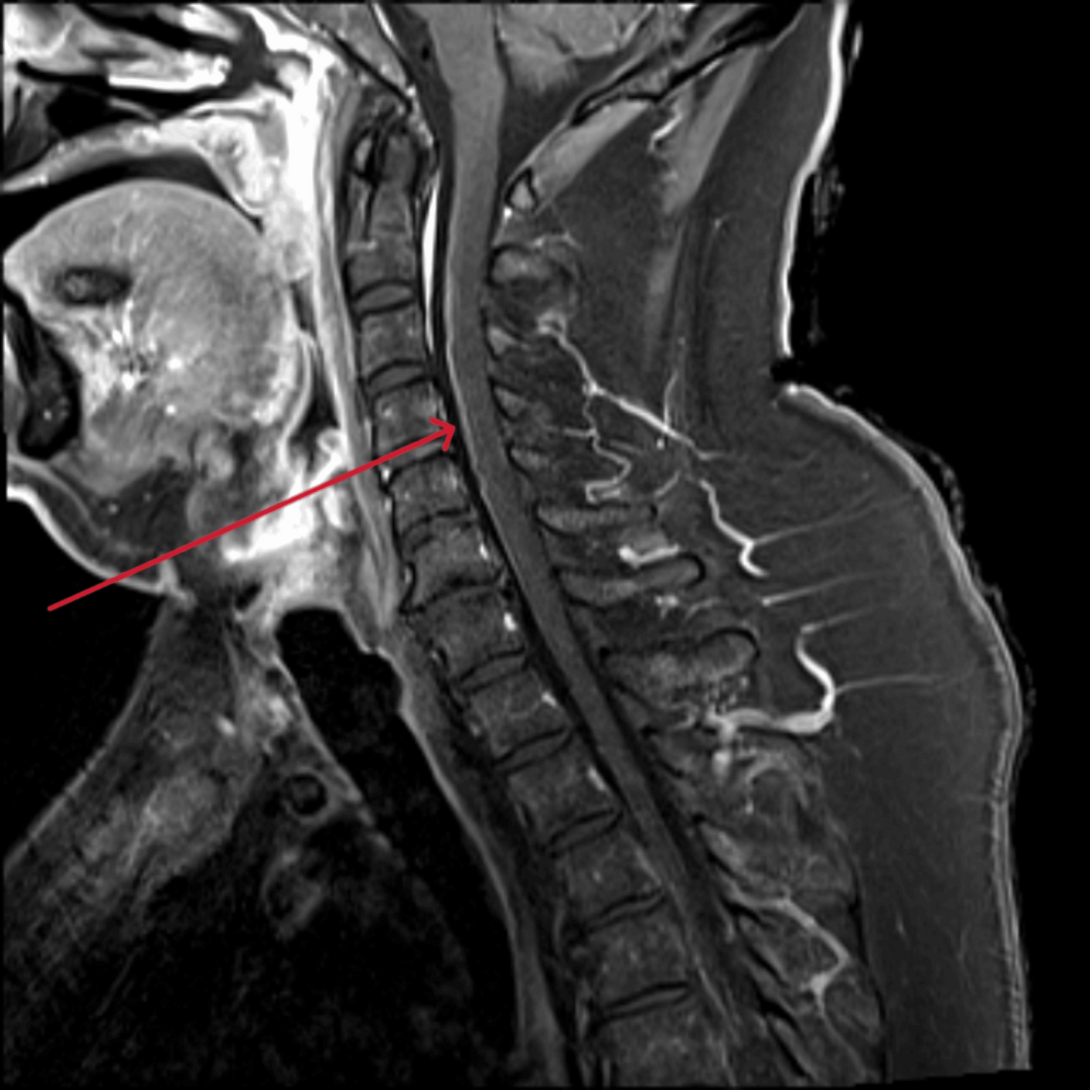
MRI showing abnormal enhancement of ventral rootlets in the cervical spine MRI: Magnetic resonance imaging

Repeat serology 14 days into admission demonstrated a fourfold rise in serum MVEV-specific IgG titer from 10 to 40, which confirmed diagnosis as per the national case definition [2]. After four sessions of alternate daily TPE, there was no evidence of meaningful neurological recovery. Repeat MRI demonstrated progressive disease involving the brain and spinal cord. There was increased FLAIR signal and swelling in the thalami bilaterally with signal change now extending into the midbrain and extensive abnormal enhancement of the ventral rootlets in the lumbar and cervical spine (Figures 3-5). A decision was made with the family to withdraw treatments 16 days into admission and our patient passed away soon after.

## Discussion

Known as Australian X disease until its isolation in 1951, MVEV is named after outbreaks centered on the Murray-Darling Basin, the largest of which occurred in 1974 [7]. It has since been found to be endemic to far northern WA and the adjacent Northern Territory, where it is maintained in a cycle between waterbirds and its main mosquito vector, *Culex annulirostris* [1].

Only a small portion of MVEV infections result in symptomatic disease; however, when clinical encephalitis does occur, it is often severe with high rates of long-term neurological sequelae and fatality [3,4]. Of the nine confirmed or presumed cases acquired in WA between March and May of 2000 reviewed by Cordova et al., there was one fatal case and three which resulted in significant neurological sequelae, including quadriparesis and brainstem impairment [4]. Complete recovery was recorded in just two cases [4]. In a review of 10 confirmed cases in WA between 2009 and 2011, Speers et al. found eight to result in either death or disability at discharge, including impaired cognition, cranial nerve palsies, and quadriplegia [3]. In the remaining two cases, a full recovery was achieved [3]. Our case therefore represents the very severe end of the disease spectrum.

MVEV is a neurotropic flavivirus in the same antigenic complex as West Nile virus (WNV), Japanese encephalitis virus (JEV), and Kunjin virus [3]. There is some evidence that MVEV may reach the central nervous system (CNS) via the olfactory route; however, given the systemic nature of the infection, it is widely believed that, like other flaviviruses, it reaches the brain via the hematogenous route following percutaneous inoculation by its arthropod vector [8,9]. The virus must then pass through the blood-brain barrier (BBB) in order to execute its neuropathogenic effects. The pathways by which it does this are still poorly understood. Some flaviviruses may cross without massively affecting the integrity of the BBB, as either cell-free virus or via infected leukocytes as part of a "Trojan horse" strategy [8]. Alternatively, infection of brain endothelial cells, coupled with the resulting inflammatory response, may induce breakdown of the BBB by cell death or tight junction (TJ) downregulation, facilitating easier viral invasion of the CNS [8].

Once the BBB has been breached, flaviviruses exhibit broad cellular tropism, infecting both neurons and glial cells [10]. Although the precise mechanisms are not entirely understood, it is likely that cell death results from a combination of direct, viral-mediated cell damage and indirect, immune-mediated neuroinflammation. JEV, dengue virus, and WNV cause cell death directly through mechanisms such as apoptosis, necroptosis, and pyroptosis [10,11]. When glial cells, particularly microglia and astrocytes, are exposed to these viruses they evoke a strong inflammatory response, upregulating inflammatory chemokine and cytokine production [10,11]. It is on this premise that TPE, an established treatment for a wide range of neuroinflammatory disorders, has been trialed for flavivirus infections refractory to other treatments and was proposed as a potentially viable option in this case [5,6,12].

MRI is the most sensitive and specific imaging modality in MVEV infection. Bilateral hyperintensity of the thalami on FLAIR or T2-weighted images is common and well-documented in the literature [1,3,13]. Indeed, early thalamic hyperintensity and involvement beyond these structures into the midbrain, as seen in our case, is associated with more severe neurological compromise and poorer outcomes [3]. Although less common, similar involvement of the cervical spinal cord, as seen on our patient’s second MRI on day 15 of admission, has also been reported [1,13]. The involvement of the lumbar spine in our patient has not previously been reported in the literature and represents a novel finding in this case. The patient’s day 15 MRI demonstrated extensive abnormal enhancement of the lumbar spine ventral rootlets, associated with discontinuous ventral cord signal change. This was thought to represent an acute demyelinating polyradiculopathy. The extent of the radiographic changes in this case correlates with the poor outcome.

Frustratingly, treatment of MVEV remains supportive, with ICU admission being required in severe cases [1]. The patient in this case was treated with dexamethasone for the first three days of his admission before it was ceased, with a further three days of intravenous methylprednisolone treatment commencing on day seven of ICU admission. Despite no studies existing to support their routine use in the context of MVEV infection, glucocorticoids are commonly used and have achieved mixed results, ranging from complete neurological recovery to permanent disability [14,15]. They may be beneficial through the reduction of intracranial pressure and their immunomodulatory effects; however, in a randomized controlled trial of 65 patients infected with JEV, treatment with high-dose dexamethasone was not found to provide any statistically significant benefit [1,16]. Similarly, there have been no trials involving antiviral drugs in the treatment of MVEV. Some studies have examined the use of certain antivirals, such as ribavirin and interferon-alpha, in cases of other flavivirus infections; however, none have proven to be fully effective [17]. Acyclovir was used in this case to cover for herpes simplex virus encephalitis and seems to be the antiviral most used in cases of MVEV infection, again with mixed results [1,15].

TPE represents a novel treatment modality used in this case. The patient underwent four alternate daily sessions in total, beginning on day eight of ICU admission. TPE removes pathogenic immune factors including autoantibodies, cytokines, and complement, and its role in the treatment of many neuroimmunological disorders is well established [12]. It was trialed in this case based on some reports of its use in other flavivirus infections. Cole et al. describe the successful use of TPE in a 10-year-old Australian boy with significant bulbar dysfunction, autonomic instability, and flaccid paralysis secondary to JEV infection [5]. Following commencement of TPE on day 16, there was improvement in power and bulbar function allowing for successful tracheostomy decannulation, and after a period of rehabilitation, this child was able to be discharged with only mild residual disability [5]. TPE has also been successfully used in a 12-year-old boy with refractory seizures secondary to WNV infection [6]. Following four sessions commencing on day eight of admission, he was seizure free and was later discharged with no neurological deficits [6].

An explanation as to why TPE proved ineffective in this case could be the delay between onset of symptoms and initiation of therapy. The family reported a prodromal illness of approximately two weeks. It is plausible that when TPE was commenced on day eight, the virus had established itself in the CNS and had already caused significant and irreversible neuronal damage, rendering TPE futile. The CSF being positive for Tau and 14-3-3 protein biomarkers may reflect this. Certain patient factors may also have contributed to the poor outcome in this case. There is a significant body of evidence to link alcohol misuse to enhanced BBB permeability, through disruption of TJ normal function and TJ degradation [18]. The patient’s significant alcohol intake of up to three bottles of wine per day may therefore have made his brain more vulnerable to the neuropathogenic effects of MVEV infection. The BBB is also known to breakdown with age [19]. Indeed, the previously discussed cases of successful flavivirus treatment with TPE were both in children [5,6]. Hills et al. report an ultimately fatal case of JEV infection in a 42-year-old adult male where TPE was tried unsuccessfully [20].

## Conclusions

This case highlights the severe neurologic sequelae that can occur secondary to infection with MVEV and underscores the frustrating lack of treatment options beyond supportive care that may be offered to these unfortunate patients. Features unique to this case include the involvement of the lumbar spine on imaging and the attempted use of TPE. Clinicians working in endemic areas, or indeed those treating travellers returning from these areas, should consider MVEV infection as a potential cause of clinical encephalitis. Although TPE proved futile in this case, its successful use in the treatment of other flaviviruses is encouraging, and its use could be considered for future cases of MVEV infection in Australia.
